# Preoperative nursing visit reduces preoperative anxiety and postoperative complications in patients with laparoscopic cholecystectomy: A randomized clinical trial protocol

**DOI:** 10.1097/MD.0000000000022314

**Published:** 2020-09-18

**Authors:** Ying Xu, Hui Wang, Meijuan Yang

**Affiliations:** Department of Vascular surgical hernia and abdominal surgery, Taizhou Hospital of Zhejiang Province, Zhejiang, China.

**Keywords:** anxiety, complication, laparoscopic cholecystectomy, preoperative nursing visit, protocol

## Abstract

**Background:**

Anxiety is a kind of emotional disorder caused by acute conditions or trigger. It is manifested in the components of the autonomic nervous system, for instance, stress, anxiety, nervosity, and discomfort. Most patients with anxiety are more active, nervous, and alert to various stimuli. Inappropriate management of early postoperative anxiety will not only prolong recovery but also increase the risk of other complications. We conduct a randomized clinical trial to investigate the influences of nursing visits against the preoperative anxiety and postoperative complications in patients undergoing laparoscopic cholecystectomy (LC).

**Methods:**

This is a single center, placebo-controlled randomized trial, which will be performed from August 2020 to December 2020. The trial is performed in accordance with the SPIRIT Checklist for randomized studies. It is authorized by the Ethics Committee of Taizhou Hospital of Zhejiang Province (D20211-34). Two hundred patients undergoing LC will be included in this study. Patients are randomly divided into 2 groups: experiential group (n = 100) or control group (n = 100). The experimental group is given preoperative nursing visit to each patient 1 day before the operation, whereas the control group did not receive the preoperative nursing intervention. The patients in experience group also received education on the surgery team and the environment of operating room, the process of anesthesia, advantages of laparoscopic surgery, and the postoperative care from recovery room to discharge. The primary outcomes include State-Trait anxiety level and postoperative visual analogue scale. Secondary outcomes include total consumption of analgesics and postoperative complications.

**Results:**

Figure (a) will show the comparison of outcomes between 2 groups.

**Conclusion:**

The preoperative nursing visit may decrease the anxiety and the complications after operation in patients receiving LC.

**Trial registration:**

This study protocol is registered in Research Registry (researchregistry5924).

## 1 Introduction

Laparoscopic cholecystectomy (LC) was conducted for the first time in 1987,^[1]^ and it is now a successful surgical method to treat the biliary colic, cholecystitis, and cholelithiasis.^[2–4]^ The popularization rate of LC is getting higher and higher, which is mainly because the surgical scars left by laparoscopic surgeries is very small; it only needs a short hospital stay, and can promote the early recovery. Compared with the traditional open surgeries, LC improves the outcome and is regarded as the standard-of-care of the cholecystitis treatment.^[5,6]^ In the United States, there are about 750,000 LCs per year, and the trend is expected to increase.^[7]^

Preoperative anxiety is a familiar phenomenon in patients undergoing preoperative assessment. It refers to the process from the start date of a specific operation to the gradually intensified process at the beginning of the operation.^[8]^ In general, it can be described as a very disturbing situation for the patients. Anxiety leads to increased pain, increased consumption of analgesics, and prolonged hospital stay, and direct impact on the medical costs.^[9,10]^ Preoperative nursing visit is one of the most effective and safest ways to offer education and psychological support for patients.^[11]^ This visit offers a chance to collect the data to better manage patients in the surgery process and to educate patients on how to cooperate with their medical care and surgical team. An informed surgical patient undergoes less anxiety and fear. Blay and Donoghue ^[12]^ have reported that the preadmission intervention of education can help decrease the levels of pain after LC, and remarkably improve the knowledge of patients’ self-care and management of complication.

However, the efficacy of preoperative nursing visits remains unclear in LC patients. We perform this randomized clinical trial protocol to study the effects of nursing visits against the preoperative anxiety and postoperative complications in patients undergoing LC. We will offer the patients with education and information about the operating room, surgical procedures, anesthesia, and preoperative and postoperative care.

## 2 Methods

### 2.1 Study design

This is a single center, placebo-controlled randomized trial that will be performed from August 2020 to December 2020. The trial is performed in accordance with the SPIRIT Checklist for randomized studies. It is authorized by the Ethics Committee of Taizhou Hospital of Zhejiang Province (D20211-34) and then is registered in research registry (researchregistry5924). The surgeon will explain the details of trial, afterward, patiently answer all the questions from patients. These patients are then presented with the written information of our trial. Each patient receives a written informed consent. As all patients participated voluntarily, they could withdraw at any time during the trial.

### 2.2 Inclusion of exclusion criteria

Candidates for inclusion are patients aged 18 to 60 years old with elective LC; ASA I-II classification; and normal platelet coagulation and count function. Exclusion criteria are as follows: patients with potential mental or physical illness; patients with the history of surgery or taking a specific drug; and severe kidney, liver, lung, and heart diseases.

### 2.3 Subjects

Two hundred patients undergoing LC will be included in this study. In the random envelope, all participants will be assigned a random number via utilizing the random number table, and the result of allocation is hidden. Patients are randomly divided into 2 groups: experiential group (n = 100) or control group (n = 100). Physicians, statisticians, data collectors, and evaluators are all blinded to the allocation.

### 2.4 Intervention

The experimental group is given a preoperative nursing visit to each patient 1 day before the operation, whereas the control group not receive preoperative nursing intervention. Preoperative anxiety and the postoperative complications, involving nausea and vomiting, and pain are then investigated for 30 minutes between the experimental group and control group. Anxiety is assessed through utilizing the Spielberger State-Trait Anxiety Inventory,^[13]^ which was translated and then proved in Iranian population. Afterwards, 2 independent psychologists examine the questionnaire for any mistranslation or bias. The questionnaire composes of 20 items and is divided into two 10-question parts to determine trait anxiety or state. It is scored from 1 (no anxiety) to 4 (the highest anxiety level) on the basis of anxiety intensity, with a total score of 20 to 80. The pain is detected utilizing the visual analogue scale. And nausea and vomiting is measured through utilizing the Johnson 10 scale criteria. The data collection involves reviewing the medical records of patients and then performing the face-to-face interviews. Patients in experimental group are interviewed the day before the operation. The interviews contain asking patients about their concerns about surgery plans and procedures, and then answering their questions in easy-to-understand phrases. The patients in experience group also receive education on the surgery team and the environment of operating room, the process of anesthesia, advantages of laparoscopic surgery, and the postoperative care from recovery room to discharge. The primary outcomes include State-Trait Anxiety level and postoperative visual analogue scale. Secondary outcomes included total consumption of analgesics and postoperative complications.

### 2.5 Statistical analysis

For data analysis, the SPSS 20.0 is employed. The patients’ descriptive characteristics are compared using the Chi-square test in the study and control groups. Paired-samples *t* test and independent samples *t* test are utilized to compare the patients’ values between the 2 groups. Variance analysis was utilized to compare the repeated measurements. A *P* < .05 is regarded the significant in statistics.

## 3 Results

**Figure 1:**
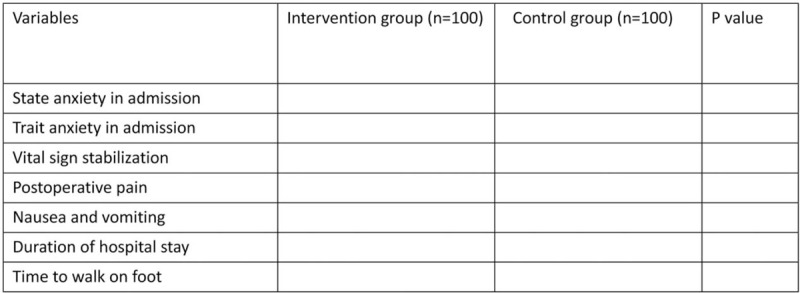
The comparison of outcomes between 2 groups.

Figure 1 shows the comparison of outcomes between 2 groups.

## 4 Discussion

The purpose of this study is to study the influences of preoperative nursing visit against the anxiety and complications after operation in the patients receiving LC. Anxiety is a kind of emotional disorder caused by acute conditions or trigger. It is manifested in the components of the autonomic nervous system, for instance, stress, anxiety, nervosity, and discomfort. Most patients with anxiety are more active, nervous, and alert to various stimuli.^[14,15]^ Inappropriate management of early postoperative anxiety will not only prolong recovery but also increase the risk of other complications.^[16,17]^ Laurion et al^[18]^ have reported that the music therapy decreased the postoperative pain, nausea, and vomiting, and reduces the pain in women undergoing laparoscopic surgery. The informative and supportive preoperative nursing visits can make patients better perceive the surgery, anesthesia, as well as rehabilitation. There is evidence that the beneficial effects of preoperative care visits on the physical recovery and complications after operation are direct rather than long-term. Although this study can provide evidence of the effect of preoperative nursing visit in patients with LC, due to the small sample size, high-quality research is still required.

## 5 Conclusion

The preoperative nursing visit may decrease the anxiety and the complications after operation in patients receiving LC.

## Author contributions

Meijuan Yang planned the study design. Hui Wang reviewed the study protocol and collected data. Ying Xu finished the manuscript. All of the authors approved the article and there is no conflict of interest.

**Data curation:** Hui Wang.

**Formal analysis:** Hui Wang.

**Funding acquisition:** Meijuan Yang.

**Writing – original draft:** Ying Xu.

## Abbreviations

Abbreviations: LC = laparoscopic cholecystectomy, VAS = visual analog scale.
